# Seasonal space use and habitat selection of GPS collared snow leopards (*Panthera uncia*) in the Mongolian Altai range

**DOI:** 10.1371/journal.pone.0280011

**Published:** 2023-01-17

**Authors:** Barry Rosenbaum, Andrey D. Poyarkov, Bariushaa Munkhtsog, Оchirjav Munkhtogtokh, Jose Antonio Hernandez-Blanco, Dmitry Y. Alexandrov, Buyanaa Chimeddorj, Bayandonoi Galtulga, Dalannast Munkhnast, Munkhtsog Bayaraa, Viatcheslav V. Rozhnov, Sebastien Comte

**Affiliations:** 1 Altai Institute for Research and Conservation, Boulder, Colorado, United States of America; 2 Laboratory of Behaviour and Behavioural Ecology of Mammals, A.N. Severtsov Institute of Ecology and Evolution, Russian Academy of Sciences, Moscow, Russia; 3 Institute of General and Experimental Biology, Mongolian Academy of Sciences, Ulaanbaatar, Mongolia; 4 Altay Branch WWF Mongolia, Khovd, Mongolia; 5 Laboratory of Population Structure, A.N. Severtsov Institute of Ecology and Evolution, Russian Academy of Sciences, Moscow, Russia; 6 WWF Mongolia Programme Office, Ulaanbaatar, Mongolia; 7 Bat Research Center of Mongolia, Ulaanbaatar, Mongolia; 8 Department of Biology, Mongolian National University of Education, Ulaanbaatar, Mongolia; 9 Wildlife Institute, College of Nature Conservation of the Beijing Forestry University, Beijing, China; 10 NSW Department of Primary Industries, Vertebrate Pest Research Unit, Orange, Australia; Universidad Miguel Hernandez de Elche, SPAIN

## Abstract

Although the home range and habitat selection of animal species is among the fundamental pieces of biological information collected by research projects during recent decades, published information on the snow leopard (*Panthera uncia*) home range is limited. The Altai Mountains of central Asia contain some of the largest and most important remaining conservation landscapes for snow leopards globally, but there is a limited understanding of the species’ ecology in this region. First, we used the data from 5 snow leopards equipped with GPS collars at four study sites in the Altai Mountains of Mongolia to broadly characterize patterns of home range use between 2013 and 2019. The data was used to calculate home range size from a 10 month period using three different estimators: minimum convex polygons (MCP), kernel density distributions (KDE), and local convex hulls (LoCoH). Second, ten data sets from 8 individual snow leopards were combined to cover all 12 months of a year and to generate a general additive mixed model of seasonal home range use and seasonal resource use. We found 1) large variation in home ranges between sites during the monitoring period ranging minimally between 26.1 and 395.3km^2^ (MCP); 2) Local convex hull home ranges were smaller compared to home ranges based on minimum convex polygons and kernels and yielded more biologically appropriate home range estimates; 3) monthly home ranges of males were larger than females; 4) female monthly home ranges decreased in summer, while male monthly range use remained stable throughout the year; and, 5) while both sexes shared similar habitat preference in winter (steep south-western slopes at high elevation), our data suggest different habitat preferences between sexes in summer. Knowledge of the space use of threatened species is crucial for their conservation, and this is especially true for apex predators who often provide benefits for an entire ecosystem. Our study provides a preliminary understanding of the spatial ecology of this important species in an area of critical conservation concern.

## Introduction

Snow leopard (*Panthera uncia*) conservation has received increasing attention in the past two decades and global interest in protecting this unique high-mountain cat continues to rise, but of the big cats, the snow leopard remains among the least studied [1, 2]. Its distribution spans 1.2–1.8 million km^2^ of high mountain habitat in 12 countries of central Asia [1–4]. Although estimates have yet to be updated, with a global population estimate of 2710–3386 mature individuals [2], the species is classified as Vulnerable by the IUCN (International Union for Conservation of Nature; http://www.iucnredlist.org), and has been given the highest legal protection in the countries where it is found [1, 2].

Despite their protected status, remaining snow leopard populations are still increasingly subject to pressure from habitat degradation and fragmentation resulting from rearing of commercial livestock. Other significant threats include mining and development, retaliatory killing in response to livestock predation, poaching for trade in fur and bones, and accidental capture in poaching snares targeted for other animals [1–6]. The impacts of climate change are also exacerbating the threats to snow leopards and their habitats and prey. Climate models estimate that by 2070 only 35% of current snow leopard range will remain as stable climate refugia and snow leopard habitats will decline by 8–23% and become increasingly fragmented [7]. Given the multitude of threats to snow leopards and their habitat, the development of conservation strategies based on reliable information on the species survival requirements is imperative. Home range and habitat use studies provide quantitative information that is essential for planning effective conservation strategies.

While ecological aspects have dominated snow leopard research, the focus has mainly been on their abundance and distribution across their range, leaving home range and habitat use among the least studied areas [2]. Knowledge of the spatial ecology of threatened species is crucial for their conservation, and this is especially true for apex predators who often provide benefits for an entire ecosystem [8, 9]. Published information on snow leopard home range is limited to 5 published studies, three of which are based on few individuals (*n* = 3–5) fitted with VHF collars [10–12], one that has used camera traps [13], and one that has used GPS technology [14]. These studies suggest that snow leopard home ranges may be significantly larger than originally estimated, which would reduce the effectiveness of current conservation strategies [14]. Home range measures are essential for understanding a species’ behavioral ecology and robust home range estimations are critical for predicting the number of animals that can be sustained within conservation areas, and for predicting long-term population persistence [14, 15].

From a conservation perspective, understanding suitable habitat for a species is critical for its future conservation [5, 16–19]. Information on whether a habitat is suitable provides insight into habitat protection, helps in identifying areas that require conservation focus, and is fundamental in determining the existence of barriers to movement and the establishment of ecological corridors in the face of colonization of former range and new areas [16–19]. Habitat modeling of snow leopards has been a frequent research topic [2]. Research in China, Nepal, and Mongolia has shown that terrain (slope, ruggedness, and aspect), elevation, and prey availability determine snow leopard habitat selection [7, 11, 14, 16, 18, 20–29]. However, habitat selection is often derived from expert opinion and from indirect signs of presence (e.g., scrapes and feces). This gives little appreciation for intensity or seasonal use of habitat and does not reflect an individual animal’s activity over space and time [30]. The advent of GPS telemetry has taken resource selection to a new level of rigor and ecological understanding [31] by providing systematic, highly accurate, and relatively unbiased data compared to indices and VHF. Availability of high-quality data on resource use has improved our ability to identify important habitats. To date only Johansson et al. [14] have published limited snow leopard habitat use data derived from GPS monitoring.

Mongolia is estimated to support the second largest numbers of snow leopards in central Asia, where approximately 1000 individuals reside [32]. More specifically, the Altai Mountains of western Mongolia are the largest continuous area of snow leopard habitat in Mongolia and believed to hold the country’s largest population [32, 33]. These mountains contain one of the largest and most important remaining landscapes globally for snow leopard conservation [5]. But there is a limited understanding of snow leopards in this region [11–14, 34–39]. Our study was motivated by the limited quantitative data on the basic spatial ecology and behavior of snow leopards in the Altai. One of the biggest challenges in conducting research on rare and elusive wildlife is small sample size. Research on snow leopards has faced this issue and many aspects of snow leopard ecology remain not fully understood because of the limited number of animals any individual study has tracked [14]. To address this limitation, we combined the data from several recent GPS studies of snow leopards in the Altai Mountains of Mongolia to broadly characterize patterns of snow leopard space use. Our objectives included estimating monthly variation in home range size and building seasonal resource selection functions. We expected 1) home ranges of males to be larger than females; 2) Female home ranges to vary seasonally but not the home ranges of males; and 3) no differences in resource use between male and female snow leopards.

## Materials and methods

### Study areas and capture methods

Our study combines tracking data of snow leopards from four study areas in the Mongolian Altai Mountains (http://whc.unesco.org/en/tentativelists/5955/). The main landscape in all four of our study areas consists of steep, rocky, broken, and dry habitats that are mainly covered in montane steppe with valley bottoms sparsely covered by arid steppe and shrubs. Higher elevations are covered by alpine vegetation and permanent snow. Altitudes range from approximately 1000–4250 m.a.s.l. The Mongolian Altai Mountains is an important habitat for endangered and vulnerable animal species such as argali sheep (*Ovis ammon*), ibex (*Capra siberica*), wolverine (*Gulo gulo*), lynx (*Lynx lynx*), snowcock (*Tetraogallus altaicus*), and golden eagles (*Aquila chrysaetos*). The Altai is occupied by semi-nomadic herders and their livestock, primarily goats (*Capra hircus*) and sheep (*Ovis aries*). Larger livestock–horses (*Equus ferrus*), yaks (*Bos grunniens*), cattle (*Bos taurus*), and camels (*Camelus bactrianus*)–are much fewer [13].

**Fig 1:**
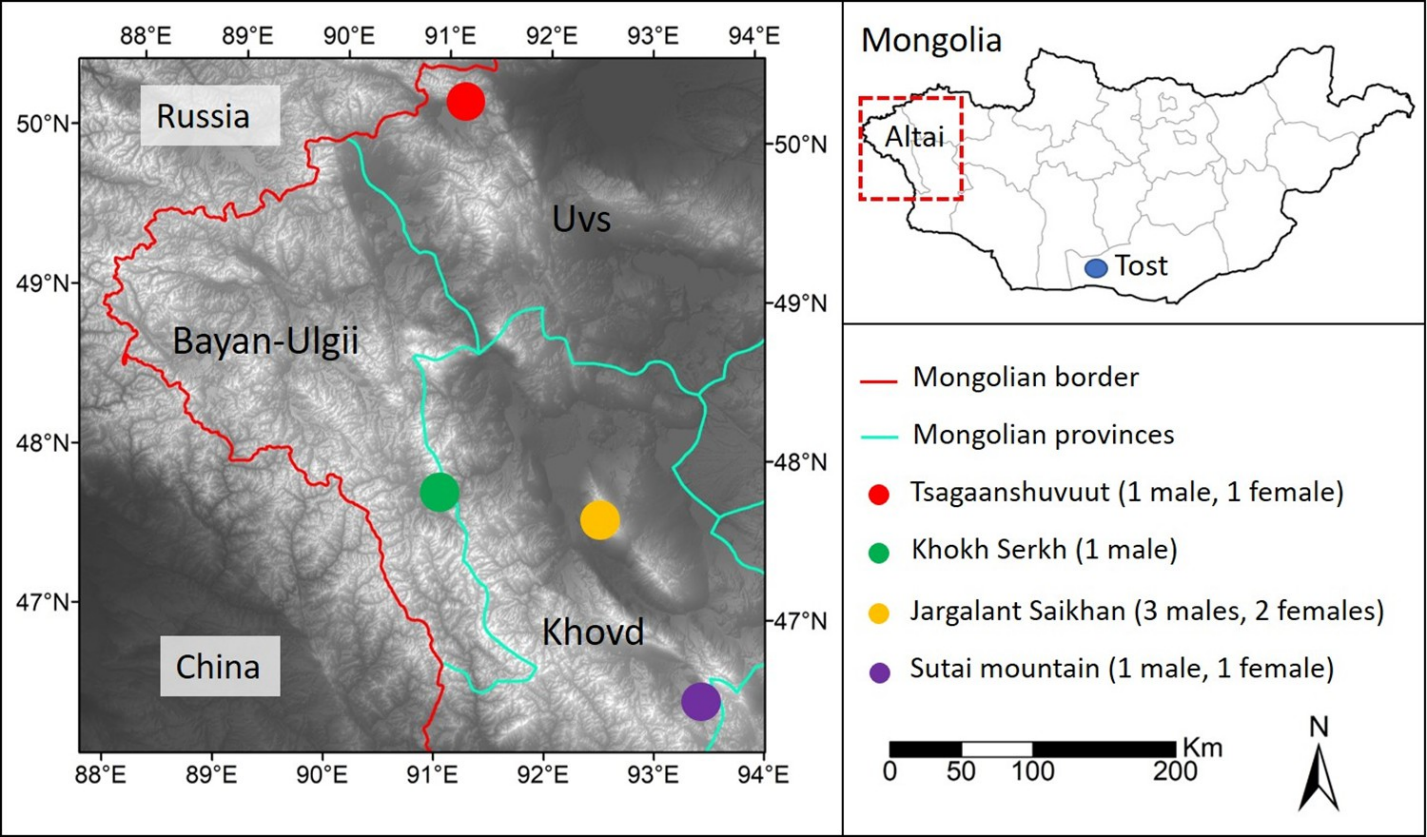
Study area locations. Locations of the four study areas in western Mongolia in Uvs, Bayan-Ulgii, and Hovd aimags. Red line indicates Mongolian national border. Light blue lines indicate aimag (provincial) borders. Background digital elevation model was created from the shuttle radar topography mission (data available from the USGS Earth Resources Observatory and Science Center:http://eros.usgs.gov/# [51].

Our most northerly site (50.29 N; 91.17 E) was located in the Tsagaan Shuvuut Strictly Protected Area of the Uvs-Nuur Biosphere Reserve, located in the Uureg-Nuur Lake basin, Uvs Province. The second site was Khokh Serkh Strictly Protected Area (KSPA), a narrow range located in Bayan Ulgii and Khovd provinces (47.81 N; 91.00 E). The third site, Jargalant Khairkhan Mountain (47.58 N; 92.58 E), is an isolated extension of the Mongolian Altai range in Khar Us Nuur National Park, Khovd province. The most southern study site (46.49 N; 93.46 E) was located in the Sutai mountain range on the border of Gov-Altai and Khovd provinces in the central part of the Mongolian Altai mountain range (Fig 1).

The protocol and procedures employed to capture and equip snow leopards with GPS collars were ethically reviewed and approved by the Russian Commission for Bioethics and the Mongolian Academy of Sciences. Capture details for each site are described in S1 File. Procedures followed closely those presented by Johansson et al. [40]. Animals were live trapped between May 2013 and August 2019 using foot snares (Table 1). Foot snares were set at snow leopard scrapes or scent sprays along cliff-base trails [12] and checked each dawn and late afternoon. Traplines were run for approximately 2–3 weeks. Upon capture, snow leopards were immobilized by intramuscular injection from an air rifle or blowpipe (Sedatives used for each individual specified in Table 1). While animals were sedated, teams monitored body condition, took body measurements, blood and hair samples, and estimated age by observation of tooth wear and examination of teats in females. Animals were equipped with GPS satellite collars (either Lotek, North Star, or Vectronics depending upon study area) and set to transmit every 1–7 hours, again depending upon study area. After immobilization reversal, animals were directly observed as they left the capture site, and then monitored in the field by VHF for 48 hours to ensure successful recovery from the sedation.

**Table 1:** Capture summary for snow leopards in the Mongolian Altai Mountains (2013–2019).

Name	Site	Sex/age	Date of capture	Capture method	Immobilization drugs †	GPS Collar ^‡^
**Tsagaana**	Tsagaanshuvuut	F/adult	10/2014	Aldrich/ Belisle	Zoletil/Dormitor/Antisedan	North Star
**Orgil**	Tsagaanshuvuut	M/adult	10/2015	Aldrich/ Belisle	Zoletil/Dormitor/Antisedan	Lotek
**SL16748**	Khokh Serkh	M/adult	10/2015	Belisle	Rompun/Ketamine HcL/ Yohimbine Hcl	Vectronics
**Sainsanaa**	Jargalant Khairkhan	M/adult	11/2016	Belisle	Ketamine Hcl / Dormitor/Antisedan	Vectronics
**Tergelsar**	Jargalant Khairkhan	F/adult	11/2016	Belisle	Ketamine Hcl / Domitor/Antisedan	Vectronics
**Nairamdal**	Jargalant Khairkhan	M/adult	10/2015	Belisle	Ketamine Hcl / Domitor/Antisedan	Vectronics
**Tsagaanbar**	Jargalant Khairkhan	M/adult	08/2019	Belisle	Ketamine Hcl / Domitor/Antisedan	Lotek
**Nairamdal**	Jargalant Khairkhan	M/adult	10/2015	Belisle	Ketamine Hcl / Domitor/Antisedan	Vectronics
**Tenger**	Jargalant Khairkhan	F/adult	5/2013; 10/2014^	Belisle	Ketamine Hcl/ Domitor/Antisedan	Vectronics
**Tsetseg**	Sutai Uul	F/adult	11/2016	§ Free capture	Zoletil/Domitor/Antisedan	Lotek
**Shuurga**	Sutai Uul	M/adult	11/2016; 10/2017^	Aldrich	Zoletil/Domitor/Antisedan	Lotek

† Zoletil (Tiletamine hydrochloride and Zolazepam hydrochloride, Virbac, France) plus 0.02 mg/kg Domitor (Medetomidine hydrochloride, Pfizer, USA). Domitor was reversed by Antisedan (Atipamezol hydrochloride, Pfifer USA) at 0.1 mg/kg; Rompun (xylazine, Bayer USA) administered at 3.0mg/kg) followed after 10 minutes by Ketamine Hcl (NexGen, USA) at 10.mg/kg. Rompun was reversed with Yohimbine Hcl (NexGen, USA) at 0.3 mg/kg; Ketamine Hcl/Domitor/Antisedan–Ketamine (3.0 mg/kg) plus Domitor (0.03mg/kg). Domitor reversed with Antisedan (0.20 mg/kg)

^‡^ Vertex Plus- Vectronics Aerospace (Germany); North Star–North Star Science and Technology (USA); LiteTrack-Lotek (Canada)

§ local inhabitants found and chased by car in open steppe valley

^ Individual caught on 2 occasions.

### Animal tracking

We combined the tracking data of ten adult snow leopards (*n* = 4 females, 6 males) in the Mongolian Altai. Two individuals, one male and one female were equipped twice with a GPS collar (Table 1) resulting in twelve individual datasets (Table 2). As our objective was to describe the spatial ecology of a “typical” adult snow leopard in the Altai, to be conservative we removed two outlier individuals from the final analysis, one male and one female (Shuurga and Tsagaana, respectively). Shuurga, with the smallest estimated home range, was recaptured at a later date and had lost his front foot, most likely in a marmot leg hold trap. Since it was unknown exactly when the foot was lost and how it affected behavior, the data was removed from the analysis. During the short time Tsagaana was tracked (3 months), she showed the greatest estimated ranging behavior (95% MCP of 770 km^2^). Although large ranging has been observed in other studies [12], we conservatively removed Tsagaana from the final analyses since our working definition of home range was based upon an area normally traversed by an animal, and excluded exploratory excursions. We did not track Tsagaana long enough to distinguish if this was a normal part of her range or exploratory behavior by a subadult (at an estimated age of 3 years she was on the border between being considered an adult or a subadult). After data formatting and subsampling, our dataset comprised 6,578 GPS locations of snow leopards with an average of 658 fixes per individual (range 83–1203).

**Table 2:** Tracking summary of snow leopards in Mongolian Altai Mountains (2013–2019) and sample sizes for space use calculations and seasonal resource selection functions.

Name	Sex	Site^†^	start	end	GPS frequency (hours)	Total GPS fixes	Success rate	GPS sample size for space use	Longterm space use^§^	Monthly home range use	Resource selection function^⸹^
Tenger	F	c	05/2013	01/2014	4	913	56	693	-	Y	w,s
Tsagaana	F	a	11/2014	02/2015	5	437	70	331	-	-	-
Sl16748	M	b	10/2015	08/2016	2^‡^	2484	82	953	Y	Y	w,s
Nairamdal	M	c	11/2015	09/2016	4	1273	66	710	Y	Y	w,s
Tenger	F	c	11/2015	11/2016	4	1728	77	1072	Y	Y	w,s
Orgil	M	a	11/2015	08/2016	1	5553	82	851	Y	Y	w,s
Tsetseg	F	d	11/2016	12/2017	1	7911	73	1203	Y	Y	w,s
Shuurga	M	d	11/2016	03/2017	4	722	90	403	-	-	-
Sainsanaa	M	c	11/2016	01/2017	7	269	87	236	-	Y	w
Tergelsar	F	c	11/2016	12/2016	7	87	67	83	-	Y	w
Nairamdal	M	c	01/2017	05/2017	4	332	76	193	-	Y	w
Tsagaanbar	M	c	08/2019	03/2020	1	2802	32	584	-	Y	w

† a = Tsagaanshuvuut; b = Khok Serkh; c = Jargalant Khairkhan; d = Sutai mountain, see Fig 1 for site locations

^‡^No fixes at 0:00, 4:00 and 8:00 am, equal to nine fixes per day.

^§^ Animals with locations available for ten months between November and September the next year

^⸹^ w = winter (November to February); s = summer (May to August)

Because the GPS collars were set in different sites by different teams, the models and the settings (i.e. duration of tracking and frequencies of fixes) differed for each individual (Table 2). The challenge was therefore to standardize the data without losing too much information so we could compare the tracking data between the different snow leopards in our study and with the available literature. We first removed any location with low quality GPS signal identified by the collars as 2D or associated with a horizontal deviation (Hdop) larger than 7 [41]. We did not have this information for two individuals (Shuurga and Tsagaana) so we visually assessed the tracking data to ensure that there were no unrealistic locations and no fixes were removed from those two already short tracking datasets. We removed all stationary locations at the beginning and the end of the tracking data for all individuals to avoid any influence of the sedation/trapping event or locations fixes collected when the collar dropped off. We standardized the tracking data following Johansson et al. [14] by randomly selecting a maximum of three locations each day. This reduced the autocorrelation between locations obtained from GPS collars while maintaining a maximum coverage of different times of the day (limited by the GPS collars settings). The final datasets were therefore as standardized as possible and met the statistical assumption of independence for the space use analyses.

### Space use analysis

The most common definition of home range refers to the area used by an animal to fulfill all its needs [42], a very difficult concept to measure for species with long life spans. Hereafter, we use the term home range use to describe the area used by an animal during a given time or define the time period of the home range. As for most long-lived species, we assumed that snow leopard home range use follows annual patterns influenced by biological and environmental seasons [14] and therefore the size of a twelve-months home range should represent a good estimation of the area needed to sustain an adult snow leopard [11]. In our study, only two individuals had a full twelve months tracking data, but five individuals had tracking data available for ten months between November and September the following year. We used those five animals to calculate a long-term home range use that could then, cautiously, be compared with annual home ranges from the literature. Following Johansson et al. [14], we used three methods to estimate the space use of snow leopards. We used the adaptive LoCoH method as it is closer to the real area used by the animals [14, 43]. After testing a range of values (around 30 km as the maximum distance between locations), we selected a = 21 km as the distance parameter [43]. We also used the minimum convex polygon (MCP) and the fixed kernel density estimator (KDE), the latter using 0.6 x href for the diffusion parameter as described in Johansson et al. [14]. All three home range estimates are presented at the 95% level.

We expected home range sizes to shrink and expand seasonally, due to both climatic conditions (mostly temperature) and biological needs (e.g. mating season, female neonatal care in den) [14, 44]. To minimize *a priori* bias about seasonality and because each animal had a different tracking duration (Table 2), we calculated the monthly space use of each individual (based on the calendar months). To assure comparable data between months, we kept only the months with a minimum of 30 locations over a minimum of 20 days. For each month, we calculated the 95% and 50% home range sizes using the adaptive LoCoH method, (a = 23 km) as it is potentially closer to the real area used by the animals [14, 49]. We used a general additive mixed model (GAMM) for each MCP level to account for nonlinear and cyclic trends across the year [45]. We therefore modeled the monthly home range use as a cyclic cubic spline (bs = “cc” in the GAMM models) with sex specific mean and shape. We added the animal ID as a random effect (bs = “re” in the GAMM models) to account for individual variations in home range sizes and for repeated measures of the same individuals. Due to the small sample size we took the assumption that year had a marginal effect. Most of the tracking data were in fact collected between October 2015 and May 2017 (Table 2).

We evaluated snow leopard habitat selection using a used/available resource selection function to describe resources that were over represented (i.e. selected) within an individual’s home range compared to their availability across a larger study area [45, 46]. To include as many individuals as possible in the analysis, and based on the space-use analyses, we subsampled the tracking into two separate four-month seasons: winter (November to February) and summer (May to August). We only included individuals for which we had tracking records across whole seasons (Table 2). For the used resources (i.e. known presence of snow leopard), we kept the same random sample of GPS locations previously used for the home range calculations. For each individual we defined the available resources for each season using a 30 km buffer around the known locations (95% MCP using all locations during the season). We then generated pseudo absence sampling points (same number as GPS locations) randomly distributed within the available area [47, 48]. The buffer size was based on snow leopard movements in our study, 30 km and included the vast majority of distances between any two GPS locations from the same animal, therefore representing the potential resources an individual could have used outside its seasonal range [47, 48].

We modeled the elevation (meters above sea level) across the study area using a 1-ha grid across the whole area calculated from the Shuttle Radar Topography Mission [49]. We then used this elevation model to calculate the slope (degrees), the aspect (0–359 degrees) and the terrain ruggedness index (positive continuous values) on each 1-ha cell using the eight neighboring cells [50, 51]. For each sampling location (used and available), we extracted the value for each covariate as the value of the cell containing the coordinates of the location. After testing for collinearity between the covariates, we removed the ruggedness index as it was strongly positively correlated with slope (Pearson’s correlation: r = 0.97). Because our sample sizes differed between seasons (i.e. different number of individuals, Table 2), we fitted two independent resource selection functions (one for winter and one for summer) using GAMMs with binomial family (0 for available resources and 1 for used resources). Slope and elevation were modelled as cubic regressions splines (bs = “cr” in the GAMMs) with sex specific means and slopes. As a circular variable, aspect was modelled as a cyclic cubic regression spline (bs = “cc”). Animal ID was included as a random effect in the models (bs = “re”). For each season, we visually present the model outputs as probabilities of use for each covariate while setting the others at their respective mean.

All home range calculations (LoCoH, MCP and KDE) were done with the package “adehabitatHR v0.4.18” [52]. The elevation data was created with the package “elevatr v0.3.1” [53]. Slope, aspect and ruggedness were derived from the elevation data using the package “raster 3.1–5” [54]. We fitted the GAMMs with the package “mgcv v1.8–3” [55]. We produced visual presentation of the model outputs with the package “ggplot2 v3.3.3” [56]. We ran all analysis using the R software v4.0.2 [57].

## Results

The home ranges calculated for the five animals with ten months of tracking data between November and September showed strong individual variations in sizes. Home ranges calculated with the LoCoH method were smaller than when using the MCP and KDE estimators (average size: 69.3 km^2^, 188.8 km^2^ and 238.2 km^2^ respectively, Table 3). This difference between estimates was especially large for the female Tsetseg with the MCP and KDE home ranges being five and six times larger than the LoCoH estimates, respectively. Unfortunately, due to the small dataset, no robust comparison between sexes or between sites could be done.

**Table 3:** Mean home range use estimates (km^2^) using 9–11 months of GPS data (see Table 2) for snow leopards (*Panthera uncia*) in the Altai Mountains, Mongolia (2013–2019).

Name	Sex	Site^†^	MCP^‡^	KDE ^§^	aLoCoH ^⸹^
sl16748	M	b	250.9	312.3	123.4
nairamdal	M	c	69.0	88.6	51.1
tenger	F	c	26.1	39.1	21.7
orgil	M	a	202.5	281.9	79.9
tsetseg	F	d	395.3	468.9	70.4

^†^ a = Tsagaanshuvuut; b = Khok Serkh; c = Jargalant Khairkhan; d = Sutai mountain

^‡^ MCP equals 95% minimum convex polygon.

^§^95% fixed kernel utility distribution, h = 0.6*href.

^⸹^ 95% adaptive local convex hull, a = 21 km.

**Fig 2:**
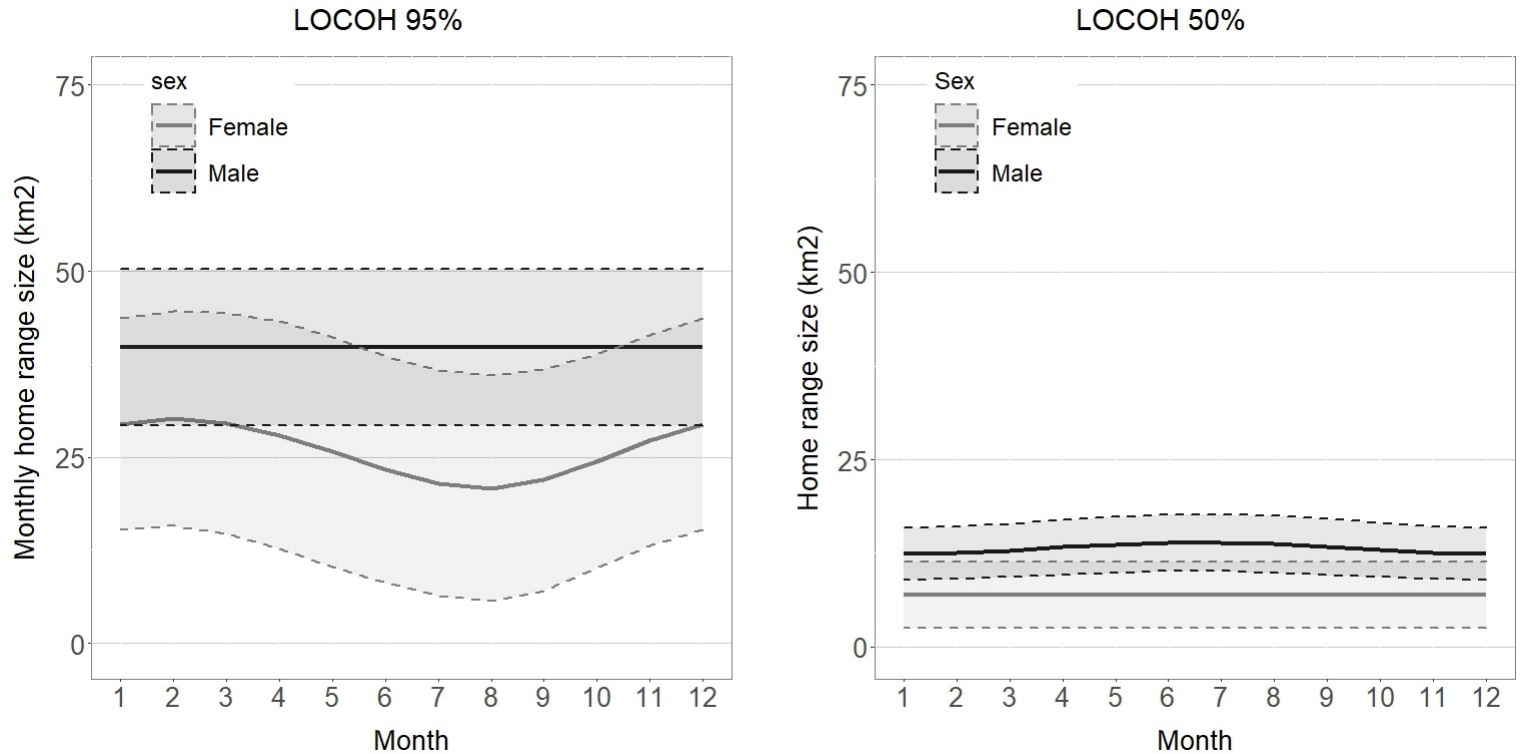
Monthly home range variation. Modeled monthly variation in snow leopard home range (n = 8; 95% MCP) using GAMM by sex and month in the Altai Mountains, Mongolia, with 95% confidence intervals (shaded).

The GAMMs fitted on the monthly home range use of eight snow leopards (three females and five males) suggest that male monthly home ranges were on average 1.88 times larger than females for the 95% LoCoH and 1.88 time larger for the LoCoH 50% (Fig 2; S1 Appendix, for full model outputs). In addition, the models showed evidence of a weak interaction between the month of the year and the female home ranges at 95% (LoCoH: edf = 1.19, p = 0.07, variance explained = 54.1%) but not at the 50% level (LoCoH: edf = 7.23E-11, p = 0.80; variance explained = 50.8%). Female monthly home range use was smallest between July and September and highest between January and March (Fig 2). There was no evidence of males having seasonal variations in monthly home range use in our study (95% LoCoH: edf = 6.75E-11, p = 0.86; 50% LoCoH: edf = 0.65, p = 0.15).

**Fig 3:**
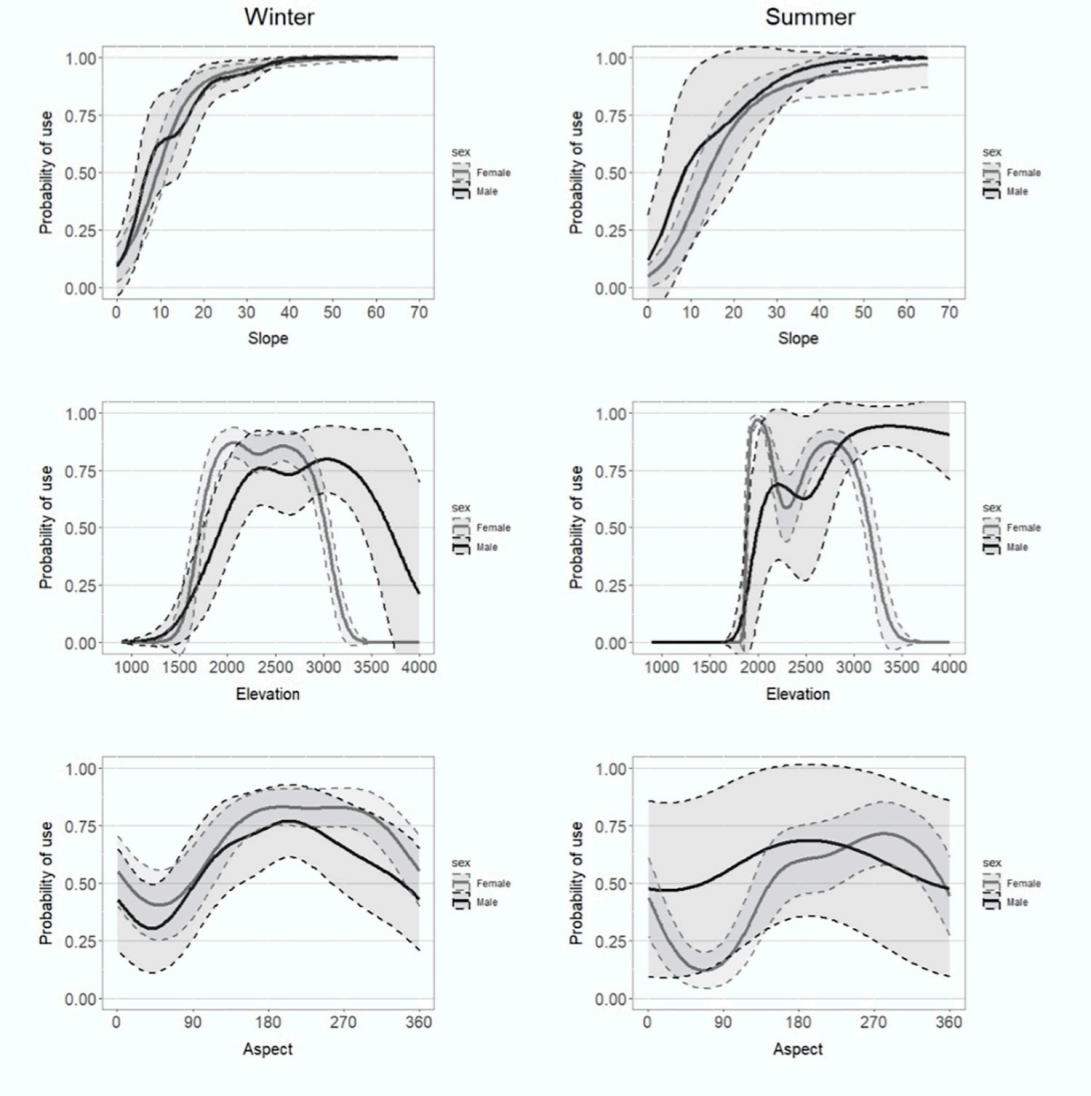
Snow leopard resource use. Modeled snow leopard habitat use (n = 8; 95% LoCoH) using GAMM in the Altai Mountains, Mongolia by sex and season (2013–2019) with 95% confidence intervals (shaded).

While eight animals (three females and five males) could be used to fit the resource selection function during winter, the sample size was limited to five individuals (two females and three males) for the summer (Table 2). In both seasons, the GAMMs showed significant interactions between sex and all three habitat covariates, while explaining 57.3% and 55.8% of the variance for winter and summer respectively (See S2 Appendix for full model output). The outputs suggest that resource selection by snow leopards in our study area varied seasonally and by sex (Fig 3). In winter, and especially in summer, areas with steep slopes were more likely to be included in snow leopard seasonal home ranges, for both sexes (Fig 3). As slope and ruggedness were strongly positively correlated in our study areas, we should also consider that seasonal home ranges of snow leopards included preferentially rugged areas. In both seasons, but more in summer, both male and female home ranges under selected areas below 2000 m. While areas above 3000m were unlikely to be included in female home range in both seasons, these high elevations areas were more likely selected in male home ranges, especially in summer. Home ranges of both sexes were more likely to include southern and western aspects in winter, a pattern kept for female home ranges in summer but not for males. In summer, aspects included in male home ranges were more evenly distributed. In general, male and females showed similar resource selection in winter but appear to select different resources in summer.

## Discussion

Our study contributes to filling a gap in our knowledge of snow leopard ecology in the Altai mountain range, which supports the population of snow leopards within Mongolia and contains some of the largest extent of habitat remaining for snow leopards globally. By combining GPS tracking data from four locations in the Mongolian Altai we 1) documented home range use; 2) found evidence that males have larger monthly home ranges than females; 3) found female monthly home range to vary throughout the year, while male monthly home range remained constant; and 4) while both sexes shared similar habitat preferences in winter (steep south-western slopes in high elevation), our results suggest differences in preferred habitat for males and females in summer.

Home range is determined by the interaction between animals and their environment, that often evolve in space and time. For example, seasonal variation in space use might be determined by the internal state of the individual and/or by changes in resource availability and distribution across the landscape [58–60]. Since these factors may change in a particular season, home range estimates calculated from data that does not include all seasons may give an underestimate of total home range use during a year. Thus, the five home ranges we estimated based upon fewer than 12 months (i.e. 9 to 11 months) and our small sample size necessitate cautious comparisons of ranging activity from other studies with data from all 12 months.

Using MCP, our five estimated home ranges for the monitored period ranged from 26.1 to 395.3km^2^. Using MCP based on VHF telemetry, McCarthy et al. [11] estimated annual home ranges for snow leopards in the Altai between 14–142 km2 (n = 5). At a different location in Mongolia, Johansson et al. [14] used GPS collars to estimate an average annual home range for snow leopards of 503 ± 286 km^2^ (n = 24). McCarthy’s overall lower estimates can be attributed to the lower number of relocations with VHF telemetry compared to the number of GPS relocations in our and Johansson’s studies [14]. The larger home range estimates from GPS data at Tost likely reflect the greater number of individuals that were studied for a longer period, resulting in a greater number of accumulated locations [14, 61–63].

MCP is widely employed in constructing home ranges despite recognition that it provides an extremely poor fit to data when the home range of an animal is strongly non-convex [64, 65]. Johansson et al. [14] found that home ranges based on adaptive LoCoH were more similar in habitat composition than those of the home ranges obtained from MCP and Kernel methods and concluded that LoCoH yielded more biologically appropriate home range estimates for snow leopards. Johansson et al. [14] estimated an average home range from aLoCoH of 179 km^2^, greater than 50% smaller than the average estimated home range he calculated using MCP. Our estimates of home range using LoCoH ranged from 22–123 km^2^. The decrease in home range was greater for larger home ranges calculated by MCP than for smaller home ranges (80–20%, respectively)–the larger the home range the greater the amount of decrease. This suggests that in large home ranges calculated by MCP much of the range is used infrequently, while snow leopards with smaller home ranges utilize their entire range more evenly.

The smallest home ranges documented in our study were from a male and female snow leopard tracked at Jargalant Khairkahn, an area supporting a potentially high density of snow leopards (Munkhtogtotkh, pers. Com). Jargalant Khairkhan is a relatively isolated mountain ridge that is separated from other mountain habitats by a wide desert and semi-desert valley with high anthropogenic use [11–14]. Although snow leopards are known to cross large expanses of unfavorable habitat [11, 14], the inhospitable terrain potentially limits the dispersal of individuals. If food resources are sufficient, individuals in confined areas may reduce their ranging behavior, which could result in increased population density. Previous studies have found an inverse relationship between home range size and population density [66–69]. Further investigations on prey availability and more robust estimates of snow leopard densities in the range are necessary to properly test this hypothesis.

Our estimates of monthly range use differed between sexes, with males using larger areas each month than females in the same study area. This difference is consistent with previous research that found snow leopards are sex-specific territorial with male snow leopards having larger home ranges than females [11–14]. Sex is an important intrinsic factor affecting the home ranges of solitary carnivores. It has been suggested that the home ranges of female large carnivores are primarily dictated by resources, but adult males in a polygynous mating system generally structure their space use to maximize mating opportunities with multiple females [70]. For the same amount of resources, adult male home range sizes therefore are usually larger than those of females to provide adult males with the possibility of overlapping with more females [70–74].

Female snow leopards in our study varied the size of their monthly home range throughout the year while space use for males was fairly constant. Female home range use decreased to the smallest extent during summer months. Females may have reduced their monthly ranging because of movement constraints imposed by reproductive behavior. None of the females collared had given birth at the time of capture, but because all were considered sexually mature because of estimated age they were considered capable of breeding during the period in which they were tracked. At least one is documented to have given birth during the study period (Tsetseg at Sutai). In Mongolia, snow leopard births occur between April and June [75]. This is the time when we documented a reduction in ranging activity. Snow leopard cubs are altricial, and the reduction in home range is likely due to young, dependent cubs limiting the mother’s mobility [60, 74, 76]. Cubs begin to following their mother from the den at 3 months [77, 78], which matches well with the time in which females begin to increase their ranging activity. Jackson and Ahlborn [79] also noted increases in home range size for a snow leopard with cubs as the young became more mobile. Marmots (*Marmota siberica*) are a main prey of snow leopards in the Altai when they become available during the spring and summer [38, 80–82], potentially providing an alternative food resource for females that support their reduction in long distance movement to find larger prey early in the summer. Interestingly, marmots are not at Tost [83] and female snow leopards do not reduce their home range use at Tost during the summer [44].

Using precise locations from GPS tracking, our data provide a robust description of habitat selection that considers individual and seasonal variations. Habitat use in our study is consistent with previous studies on snow leopards showing that both males and females select for similar terrain [16, 21, 22, 24, 27, 28, 84], preferring higher elevations (2500–4000 m) with steep slopes (20–60°) and a generally southwest exposure (180° – 270°). Although subtle, some seasonal differences in habitat selection between males and females were apparent in our study. Female snow leopards in summer showed stronger selection for the steepest slopes, which was associated with high terrain ruggedness. These are ideal places to rest and for females with cubs to use for dens [85] as they provide a good view of the surrounding terrain to spot potential prey or threats.

Females showed strong avoidance of easterly and to lesser extent southerly slopes in summer. Bai et al. [24] concluded that exposure had little influence on snow leopard habitat preference; but the results were based on static signs of presence (e.g., scats, marking sites, etc.) and did not distinguish among intensity of use, season, or sex. Both females and males showed a preference for south facing slopes in winter. Xu et al. [22] also found a preference by snow leopards for southerly exposures in winter. South facing slopes evaporate snow more quickly and the large ungulates upon which snow leopards prey forage on the exposed vegetation in these areas in winter [86]. This preference may also aid in thermoregulation.

In one of the earliest papers on snow leopard ecology, Hemmer [77] reported that snow leopards may make regular seasonal movements from one part of their individual range to another, and that the seasonal movement may depend upon climatic conditions and the movements of ungulate herds. Seasonal elevation movements have also been reported for snow leopard populations in India and Kyrgyzstan [20, 87]. In our study areas male snow leopards were more likely to use higher elevations than females during the summer. Examinations of prey kill sites indicate that both male and female snow leopards preferentially prey on ibex and argali males [91]. Adult male ibex and adult male argali segregate from females in the summer, using the more moderate slopes at higher elevations [88–91]. The pattern we notice of male snow leopards using higher elevations may indicate that male snow leopards are able to follow males at this time while female are constrained to the lower, but steeper and more rugged, hillsides in summer to care for young cubs. Temperatures at higher elevations are also cooler with more frequent breezes, thus potentially reducing the effect of higher summer temperatures on males.

The study of spatial ecology is needed to inform the design of management and conservation interventions, particularly for those species that are scarce and lead secretive lives. Due to their large ranging behavior and the remote areas in which they live, snow leopards in the Mongolian Altai are difficult animals to capture and fit with a GPS collar and few studies have been done. By combining the tracking data from projects at four different sites we were able to give cautious minimum estimates of home range use by adult snow leopards, show sex specific seasonal trends in snow leopard space and resource use. The volume of location data obtained by GPS technology is appropriate for individual level questions, but the data from several animals, within the limitations presented by different locations and different times, allows us to answer some population level questions about general space use. In the face of future threats, this new information on of the spatial ecology of this vulnerable species in an area of global conservation concern [5] is important for its conservation as an apex predator that provides benefits for an entire ecosystem.

## Supporting information

S1 Appendix Monthly home range use—model outputs. (DOCX)

S2 Appendix Resource selection functions—model outputs. (DOCX)

S1 File Capture details. (DOCX)
