# Supplementary material for: Seasonal space use and habitat selection of GPS collared snow leopards (Panthera uncia) in the Mongolian Altai range
Source: PLoS One. 2023 Jan 17;18(1):e0280011. doi: 10.1371/journal.pone.0280011 (PMC10045553; doi:10.1371/journal.pone.0280011)
Supplement: S1 Appendix — (DOCX) [file pone.0280011.s001.docx]

# **S1 Appendix: Monthly home range size – model outputs**

| **Local Convex Hub (LoCoH) 95%:** | |  |  |  |
| --- | --- | --- | --- | --- |
| *Parametric coefficients* | |  |  |  |
|  | **Estimates** | **Std error** | **t-value** | **p-value** |
| Intercept | 26.516 | 7.051 | 3.761 |  |
| Males | 13.281 | 8.866 | 1.498 | 0.14 |
|  |  |  |  |  |
| *Smooth terms* |  |  |  |  |
|  | **edf** | **Ref edf** | **F test** | **p-value** |
| Month : females | 1.187 | 2 | 6.843 | 0.07 |
| Month : males | 6.745 e^-11^ | 2 | 0.000 | 0.86 |
|  |  |  |  |  |
| Adjusted R^2^ | 0.498 |  |  |  |
| Deviance explained | 54.1% |  |  |  |
|  |  |  |  |  |

| **Local Convex Hub (LoCoH) 50%:** | |  |  |  |
| --- | --- | --- | --- | --- |
| *Parametric coefficients* | |  |  |  |
|  | **Estimates** | **Std error** | **t-value** | **p-value** |
| Intercept | 6.913 | 2.239 | 3.087 |  |
| Males | 6.126 | 2.820 | 2.172 | 0.03 |
|  |  |  |  |  |
| *Smooth terms* |  |  |  |  |
|  | **edf** | **Ref edf** | **F test** | **p-value** |
| Month : females | 7.234 e^-10^ | 2 | 0.000 | 0.800 |
| Month : males | 0.654 | 2 | 0.713 | 0.152 |
|  |  |  |  |  |
| Adjusted R^2^ | 0.467 |  |  |  |
| Deviance explained | 50.8% |  |  |  |
