# Supplementary material for: Seasonal space use and habitat selection of GPS collared snow leopards (Panthera uncia) in the Mongolian Altai range
Source: PLoS One. 2023 Jan 17;18(1):e0280011. doi: 10.1371/journal.pone.0280011 (PMC10045553; doi:10.1371/journal.pone.0280011)
Supplement: S2 Appendix — (DOCX) [file pone.0280011.s002.docx]

S2 Appendix: Resource selection functions – model outputs

| **2a. Winter (November – February):** | |  |  |  |
| --- | --- | --- | --- | --- |
| *Parametric coefficients* | |  |  |  |
|  | **Estimates** | **Std error** | **z-value** | **p-value** |
| Intercept | -1.304 | 0.534 | -2.442 |  |
| Males | 0.545 | 0.610 | 0.894 | 0.371 |
|  |  |  |  |  |
| *Smooth terms* |  |  |  |  |
|  | **edf** | **Ref edf** | **Chi-square** | **p-value** |
| Elevation : females | 5.976 | 6.845 | 109.1 | <0.001*** |
| Elevation : males | 5.537 | 6.537 | 104.5 | <0.001*** |
| Slope : females | 2.863 | 3.565 | 282.8 | <0.001*** |
| Slope : males | 5.791 | 6.551 | 378.4 | <0.001*** |
| Aspect : females | 4.010 | 8.000 | 103.8 | <0.001*** |
| Aspect : males | 5.361 | 8.000 | 132.9 | <0.001*** |
|  |  |  |  |  |
| Adjusted R^2^ | 0.631 |  |  |  |
| Deviance explained | 57.3% |  |  |  |
|  |  |  |  |  |

| **2b. Summer (May – August):** | |  |  |  |
| --- | --- | --- | --- | --- |
| *Parametric coefficients* | |  |  |  |
|  | **Estimates** | **Std error** | **z-value** | **p-value** |
| Intercept | -39.821 | 5.935 | -6.710 |  |
| Males | 36.271 | 6.302 | 5.756 | <0.001*** |
|  |  |  |  |  |
| *Smooth terms* |  |  |  |  |
|  | **edf** | **Ref edf** | **Chi-square** | **p-value** |
| Elevation : females | 4.993 | 5.000 | 97.1 | <0.001*** |
| Elevation : males | 4.551 | 4.803 | 123.5 | <0.001*** |
| Slope : females | 2.394 | 2.914 | 179.0 | <0.001*** |
| Slope : males | 3.484 | 3.931 | 154.5 | <0.001*** |
| Aspect : females | 3.785 | 4.000 | 111.3 | <0.001*** |
| Aspect : males | 2.192 | 4.000 | 24.4 | <0.001*** |
|  |  |  |  |  |
| Adjusted R^2^ | 0.619 |  |  |  |
| Deviance explained | 55.8% |  |  |  |
