# Supplementary material for: Seasonal space use and habitat selection of GPS collared snow leopards (Panthera uncia) in the Mongolian Altai range
Source: PLoS One. 2023 Jan 17;18(1):e0280011. doi: 10.1371/journal.pone.0280011 (PMC10045553; doi:10.1371/journal.pone.0280011)
Supplement: S1 File — (DOCX) [file pone.0280011.s003.docx]

Capture details

The Altai Mountains of Mongolia (http://whc.unesco.org/en/tentativelists/5955/) constitute a major and central part of the Altai Mountain range located at the junction of Central Asia and Siberia (Fig 1). The Altai has many summits near or exceeding 4000 meters above sea level (m asl) and stretches for some 900 kilometers from the north-western boundary of the country to the south, through Bayan-Ulgii, Uvs, and Khovd provinces. Over 20 peaks are capped with permanent snow fields. The high ridges of the Altai descend to large basins and dry steppes that extend eastward across vast areas. The Altai contains the complete sequence of altitudinal vegetation zones found in central and east Asia, from arid steppe, steppe, forest-steppe, montane steppe, mixed forest, subalpine vegetation to alpine vegetation. The range is important habitat for endangered and vulnerable animal species such as the snow leopard, argali sheep (*Ovis ammon ammon*), ibex (*Capra siberica*), wolverine (*Gulo gulo*), lynx (*Lynx lynx*), snowcock (*Tetraogallus altaicus*), and golden eagles (*Aquila chrysaetos*).

The protocol and procedures employed at each site were ethically reviewed and approved by the Russian Commission for Bioethics and the Mongolian Academy of Sciences. Procedures followed closely those presented by Johansson et al. [92]. The most northerly study site was located in the Tsagaan shuvuut Strictly Protected Area of the Uvs-Nuur Biosphere Reserve, located in the Sagil soum of the Uureg-Nuur Lake basin, Uvs Province (Fig 1). The main landscapes include montane steppes and alpine vegetation. The maximum elevation at the study site is 3496 m asl. Snow leopard trapping was conducted in the autumns of 2014 and 2015 using Aldrich and Belisle 8" foot snares (Goodrich *et al.* 2010). Body weight was estimated visually and snow leopards were then immobilized with an intramuscular injection of 2.5 mg/kg of Zoletil (Tiletamine hydrochloride and Zolazepam hydrochloride, Virbac, France) mixed with 0.02 mg/kg Domitor (Medetomidine hydrochloride, Pfizer, USA). Domitor was reversed by Antisedan (Atipamezol hydrochloride, Pfifer USA) with a dosage of 0.1 mg/kg. A female snow leopard (Tsagana) weighing 31 kg was caught in October 2014 in the Khoid Sair gorge. Her age was estimated at three years old, classifying her as a subadult, and based on the examination of the nipples, it was concluded that she had never given birth. The female was collared with a GPS satellite transmitter from North Star Science and Technology (USA). The transmitter was programmed to transmit the coordinates of the animal's location every 5 hours. One adult male (Orgil) weighing 36 kg was caught in the Omno Sair gorge in October 2015, sedated using the same protocols used the year before, and was fitted with a Lotek GPS-Iridium collar (Canada) equipped with a drop-off system. The satellite GPS transmitted coordinates every hour.

Our second site was Khokh Serkh Strictly Protected Area (KSPA) area, a narrow range located in Bayan Ulgii and Khovd provinces (Fig 1). Altitudes range from 2500m to 4019m a.s.l. The Protected Area consists of steep, rocky, and dry habitat and is mainly covered in montane steppe with valley bottoms sparsely covered by arid steppe and shrubs and higher elevations covered by alpine vegetation. In October 2015 a 40 kg male (approximately 4 years old by teeth examination) snow leopard (SL16748) was caught in Baga Yaamat valley using a Belisle 8” foot snare. The animal was sedated with xylazine (3.0 mg/kg) followed by ketamine (10.0 mg/kg) after ten minutes. The xylazine was reversed with yohimbine (0.3 mg/kg). The male was collared with a Vertex Plus collar from Vectronics Aerospace (Germany) programmed to transmit a location every 2 hours.

The third site was Jargalant Khairkhan Mountain, an isolated extension of the Mongolian Altai range in Khar Us Nuur National Park, Hovd Aimag. It has a summit elevation of 3,796 m a.s.l. Habitats vary from high-mountain tundra, alpine meadows and high-mountain steppe to arid steppe. Snow leopards were captured using 8”- Berlisle foot snares and sedated using ketamine and medetomidine. Sedation was reversed by atipamezole. A female (Tenger) was captured in May 2013 and fitted with a Vertex Plus collar (Vectronics Aerospace, Germany). She was recaptured on October 9, 2014, and the collar was replaced with another Vertex Plus transmitter. A male (Nairamdal) was captured in October 2015 and fitted with a Vertex Plus collar (Vectronics Aerospace, Germany). A male (Sainsanaa) and female (Tergelsar) were collared in November 2016 with Vertex Plus collars, and another male (Tsagaanbar) was collared in August 2019 and fitted with a LiteTrak Iridium 420 (Lotek LLC, Canada).

Our most southern study site was located in the Sutai mountain range on the border of Gov-Altai and Khovd provinces (Fig 1). Its highest point, Sutai-Khairkhan (4,250 m a.s.l.), is the fourth highest peak of the Mongolian Altai Mountains. The upper part of the Sutai-Khairkhan summit is covered with permanent snow. In November 2016, local inhabitants found and chased a female snow leopard (Tsetseg) by car in an open steppe valley southwest of Tsetseg Nur. The 30 kg female ran 100 - 300 meters and then laid down in vegetation. It was possible to approach the female to administer a sedation dart by blowpipe. The sedation technique was the same as used at the first described study site. Inspection of the nipples indicated that the three year old female had never given birth. The female was fitted with a Lite track Lotek Iridium track M collar programmed to fix one location per hour. An adult male (Shuurga), weighing 46 kg, was caught in a side gorge overlooking the valley to Lake Tsetseg on November 12, 2016, using an Aldrich's foot snare. The immobilization was carried out using the sedation technique used in the Tsagaan shuvuut Strictly Protected Area study. The male leopard was fitted with a Lotek GPS Argos GPS satellite collar that transmitted a location every 4 hours. The collar worked until March 26, 2017. The animal was recaptured on October 23, 2017. It had lost the distal part of its front left paw, likely as a result of being caught in a trap set for marmots.
